# Retinoic acid-related orphan receptor alpha (RORα) as a candidate multi-pathway target in Alzheimer’s disease: mechanisms and therapeutic prospects

**DOI:** 10.3389/fnins.2026.1912268

**Published:** 2026-08-20

**Authors:** Shivakanth Chintalapally, Kalpana Rajanala, Arun Upadhyay

**Affiliations:** 1 Ocugen India, Hyderabad, Telangana, India; 2 Ocugen, Malvern, PA, United States

**Keywords:** AAV vectors, Alzheimer’s disease, amyloid precursor protein, gene therapy, neuroinflammation, neuroprotection, *RORA* gene, RORα protein

## Abstract

The development of Alzheimer disease (AD) involves a cluster of pathogenic processes, including amyloid-beta (Aβ) deposition, tau-mediated neurodegeneration, chronic neuroinflammation, oxidative stress (OS), metabolic dysregulation, and disruption of circadian rhythms. Nuclear hormone receptor, Retinoic Acid-Related Orphan Receptor Alpha (RORα) was shown to regulate multiple neuroprotective pathways such as inflammatory signaling (NF-κB suppression), mitochondrial integrity and mitophagy, redox homeostasis [upregulation of glutathione peroxidase 1 (GPX1), and mitochondrial superoxide dismutase 2, (SOD2)], calcium-dependent synaptic architecture [inositol 1,4,5-trisphosphate receptor type 1 (ITPR1), Purkinje cell protein 4 (PCP4)], and circadian rhythm stability [period 2 (PER2), brain and muscle ARNT-like 1 (BMAL1)]. Multi-omics network analyses place RORα within regulatory networks that are co-associated with key AD-related genes and supports an associational, network-based relationship for *RORA*. Preclinical gene-augmentation studies using adeno-associated viral vectors report that RORα overexpression reduces APP levels, remodels the complement regulator CD59 glycoprotein (CD59), inhibits OS, and enhances neuronal survival, although these effects were established largely in retinal and other non-AD systems. These findings support the potential of *RORA* as a therapeutic target through genetic intervention, but direct demonstration of AD-modifying efficacy *in-vivo* is still lacking. Investigational *RORA*-focused gene therapy in retinal degenerative diseases provides proof-of-concept for, but does not yet establish, applicability within the central nervous system. Taken together, this evidence nominates *RORA* as a candidate system-level regulator that may help restore disrupted homeostatic transcriptional networks in AD, a hypothesis that remains to be tested. We propose that RORα functions as a transcriptional hub coupling three homeostatic axes that fail in AD; the circadian, mitochondrial-metabolic, and immune-inflammatory axes, and that its regional expression changes in AD (hippocampal up-regulation vs. suprachiasmatic down-regulation) represent a compensatory response that ultimately fails. Cell-type-specific expression profiling is required to determine in which regions augmentation may be therapeutically appropriate. Restoring RORα is therefore could be network-stabilizing rather than single-pathway intervention.

## Introduction

**FIGURE 1:**
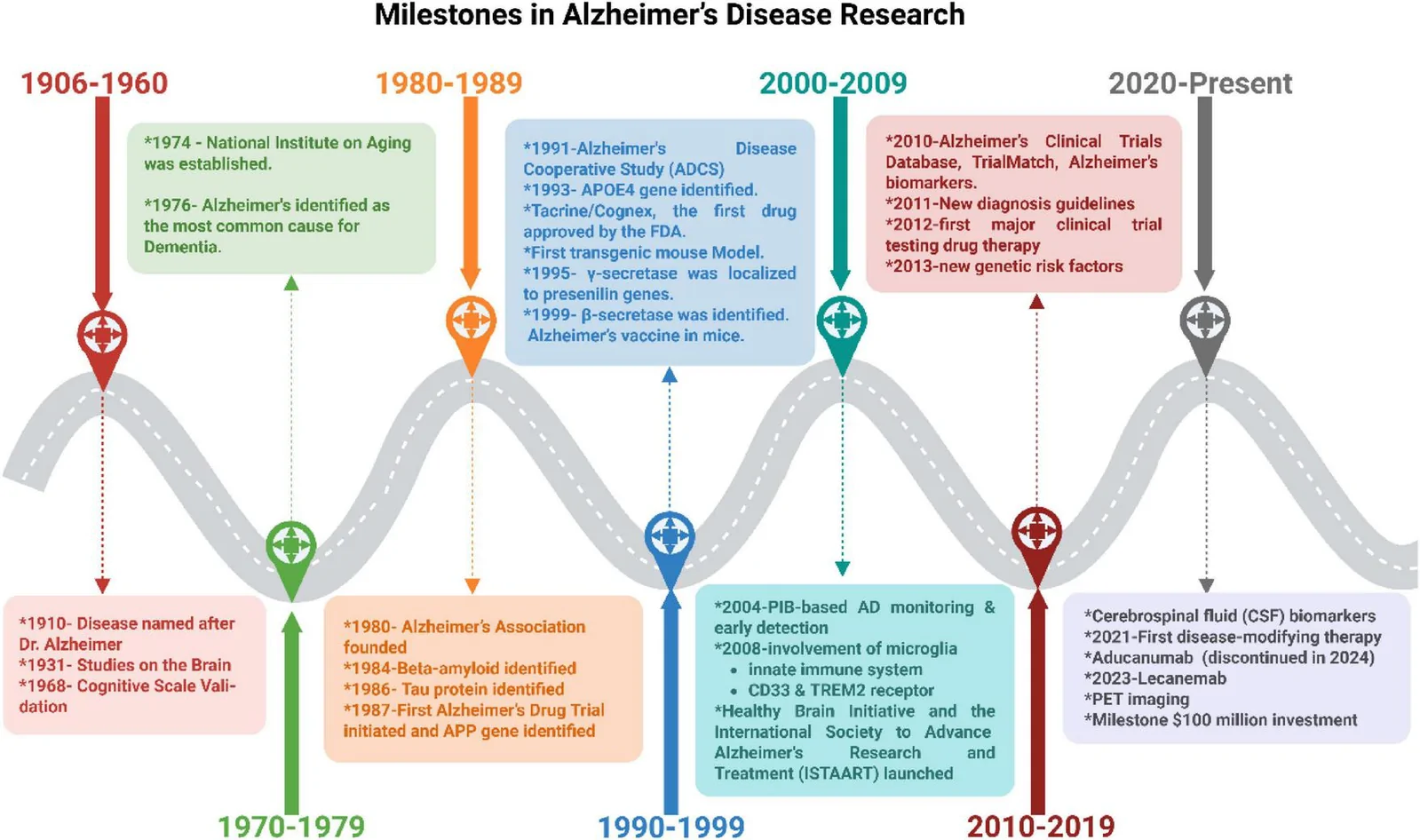
Timeline of research in AD. Between 1906 and 1960 the disorder was first formally described along with original neuropathological studies providing the groundwork of pathological basis. The next two decades, starting with the 1970s, saw the development of national research efforts, the official recognition of AD as the most common etiology of dementia, and the discovery of primary genetic susceptibility factors, such as APP, APOE4, and presenilin alleles, and finally, the first disease-modifying therapeutic agent was approved by the FDA. Simultaneously, the field has promoted the research of biomarkers by means of the analysis of cerebral spinal fluid assessment tools and positron emission tomography visualization methods; it streamlined the structure of clinical trials and acknowledged the auxiliary role of neural immune processes. The era of the 2020s has seen the emergence of the actual disease-modifying therapeutics that can serve as the embodiment of decades of mechanistic knowledge. Together, these accomplishments shed light on the gradual clarification of the AD pathobiology and the continuing application of research findings in the laboratory to the diagnostic and therapeutic invention.

Dr. Alois Alzheimer, a German physician, first described Alzheimer’s disease (AD) in 1906 after he discovered twisted fiber bundles and unusual aggregates in the brain cells of women with the disease (Alzheimer, 1907). Since then, researchers have expanded our understanding of how AD affects the brain and how to help those affected and their families. Some important milestones in the AD research timeline are depicted below in Figure 1 (Alzheimer’s Association, 2025). AD, which is a complex, progressive neurodegenerative disorder, is the leading cause of dementia worldwide (WHO, 2017).

## Epidemiology and etiology

AD is the leading cause of dementia worldwide. Globally, an estimated 55–57 million people lived with dementia as of 2021, of which AD accounts for approximately 60–70%, with around 10 million new cases each year, and over 60% of affected individuals live in low- and middle-income countries (WHO, 2025). This total is projected to reach roughly 139 million by 2050 (Daci and Flotte, 2024). Collectively, these data underscore the accelerating global and national burden of AD, driven primarily by population ageing and the absence of curative interventions.

## Molecular biology and pathophysiology

The cholinergic system, amyloid, tau protein, inflammation, OS, metal ions, glutamate excitotoxicity, the microbiota-gut-brain axis, and abnormal autophagy are some of the concepts that have shaped our understanding of AD disorders. However, further research and validation are required to identify the exact mechanisms by which known causes and risk factors lead to AD and understand how its clinical features relate.

The causes of AD may include a lack of acetylcholine, neuroinflammation, OS, dysregulation of glutamatergic neurotransmission, insulin resistance, gut microbiome dysregulation, disruptions in cholesterol balance, mitochondrial dysfunction, and impaired autophagy.

**FIGURE 2:**
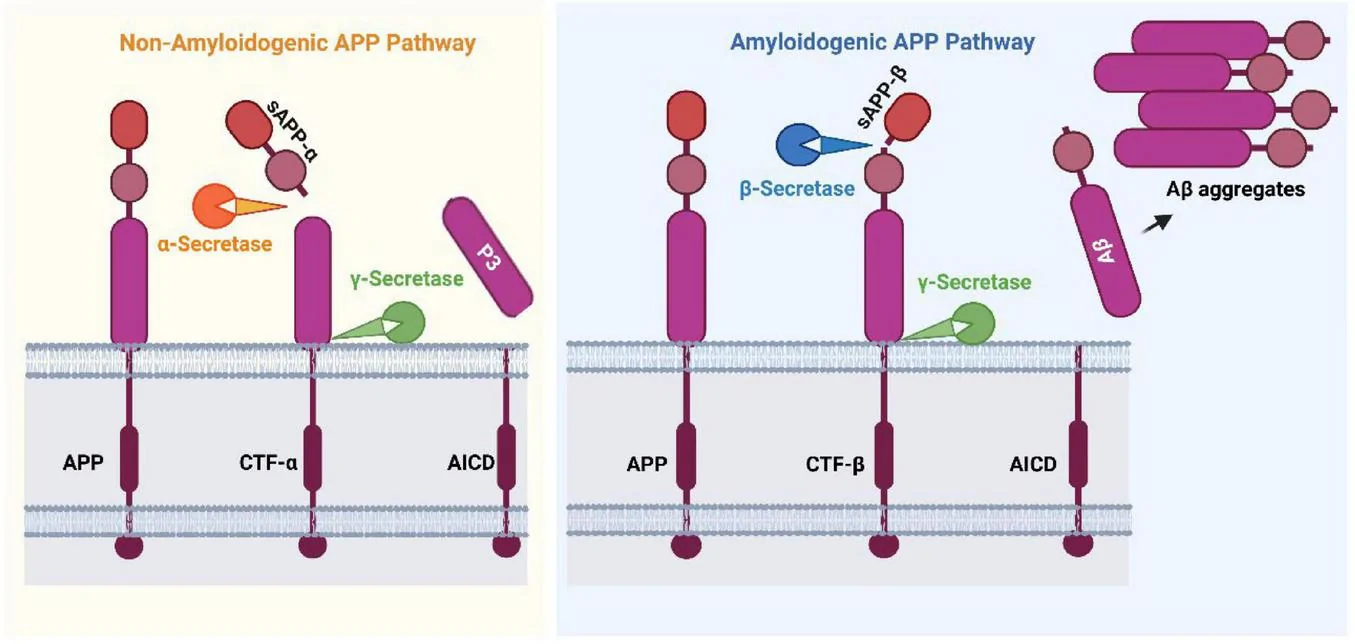
Amyloid precursor protein (APP) pathway. The non-amyloidogenic APP pathway incorporates α-secretase followed by γ-secretase, while the amyloidogenic APP pathway engages β-secretase followed by γ-secretase, resulting in the formation of Aβ aggregates, a characteristic of AD. Created in https://BioRender.com.

In humans, APP undergoes alternative proteolytic processing via amyloidogenic (AG) and non-amyloidogenic (NAG) routes that differentially govern Aβ generation (Figure 2). In the NAG pathway, membrane-bound α-secretases sequentially process APP (Chen et al., 2017). They cleave within the Aβ domain to produce the membrane-tethered α-C terminal fragment CTFα (C83) and the N-terminal fragment sAPPα. After that, γ-secretases cleave CTFα to produce the APP intracellular domain (AICD) and extracellular P3 domain (Figure 2). In the AG pathway, APP undergoes processing by membrane-bound β- and γ-secretases in a particular sequence. When β-secretase cleaves APP, it produces the membrane-tethered C-terminal fragment β (CTFβ or C99) and the N-terminal sAPPβ. Subsequently, γ-secretases cleave CTFβ into the intracellular domain of APP (AICD) and extracellular Aβ domain (Figure 2) which leads to the formation of Aβ aggregates. When AD-related Aβ levels were experimentally altered, PP1 levels and activity changed, which affected the tau balance (Lagomarsino et al., 2021). In some AD mouse models, such as APP/PS1KI or 5XFAD, the endogenously produced murine apolipoprotein E (ApoE) undergoes proteolytic processing that generates distinct ApoE fragments (Huang and Mahley, 2014; Saul and Wirths, 2017). The resulting cleavage pattern closely parallels the pathological fragmentation of human ApoE observed in AD, with particular relevance to the ApoE4 isoform (Harris et al., 2003; Huang and Mahley, 2014). Because ApoE proteolysis has been associated with synaptic dysfunction, neurotoxicity, and amyloid-related pathology, these models more accurately reproduce ApoE-dependent pathogenic mechanisms seen in human AD than models in which murine ApoE remains intact (Harris et al., 2003) When tau oligomers or Aβ activate indoleamine-2,3-dioxygenase 1 (IDO1), tryptophan is converted to kynurenine, which inhibits astrocytic glycolysis and reduces key source of energy for neurons leading to synaptic dysfunction and neurodegeneration (Minhas et al., 2024). Inhibition of IDO1 improves cognition in several animal models of AD and restores synaptic plasticity *in-vitro* (Minhas et al., 2024).

### Astrocytes in AD

The regional diversity of astrocytes in the aging brain shows how complex their responses become after chronic exposure to AD neuropathologic change (Acquaah-Mensah et al., 2015). Distinct patterns of astrocytic reactions may be linked to Aβ plaques and pTau. A five-region snRNA-seq study of astrocytes from 32 donors across the normal aging to severe AD continuum revealed the existence of intermediate states that appear to be transitional between homeostatic and reactive astrocytes (Serrano-Pozo et al., 2024).

### Neuroinflammation

**FIGURE 3:**
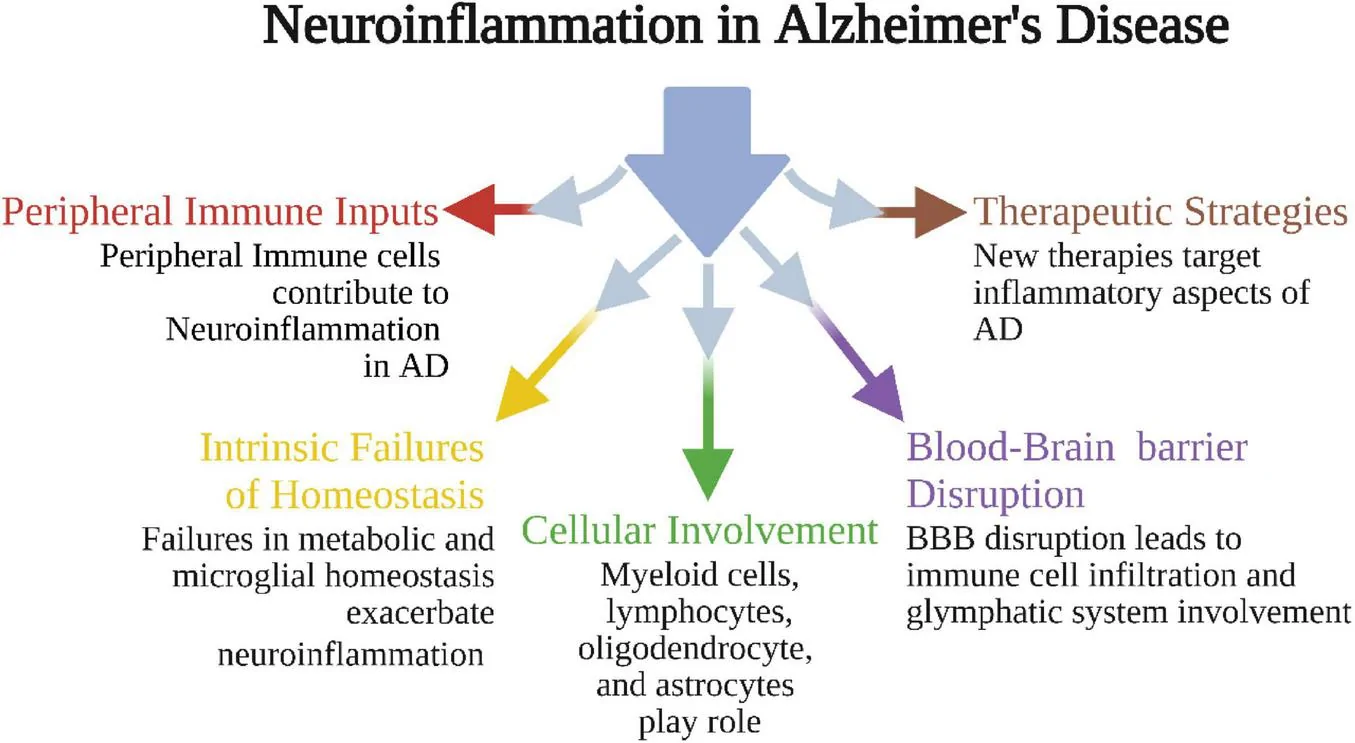
Neuroinflammation in AD. This figure shows a systematic, systems-wide representation of the contributory factors to, and ramifications associated with, neuroinflammation in AD. The illustration clarifies the complex interrelated mechanisms of signal transduction pathways that sustain chronic inflammation in the brain affected by AD: *Peripheral Immune Inputs (Orange)*: Systemic immune cells contribute to the establishment and regulation of the inflammatory milieu within the central nervous system. *Intrinsic Failures of homeostasis (Yellow)*: Impaired metabolic and microglial homeostasis exacerbate the inflammatory response. *Cellular Involvement (Green)*: Lists the type of cells that are involved in the inflammatory process, and it includes myeloid cells, lymphocytes, oligodendrocytes and astrocytes. *Blood-Brain Barrier (BBB) Disruption (Purple)*: BBB damage leads to higher immune cell invasion and the impairment of the functionality of the glymphatic system. *Therapeutic Strategies (Brown)*: Overview of the development of new therapeutic intervention based specifically on these inflammatory mechanisms, which could be a potential route of treatment of AD. Created in https://BioRender.com.

Neuroinflammatory pathology in AD is driven by interacting peripheral immune inputs and intrinsic failures of metabolic and microglial homeostasis (Heneka et al., 2025; Figure 3).

**FIGURE 4:**
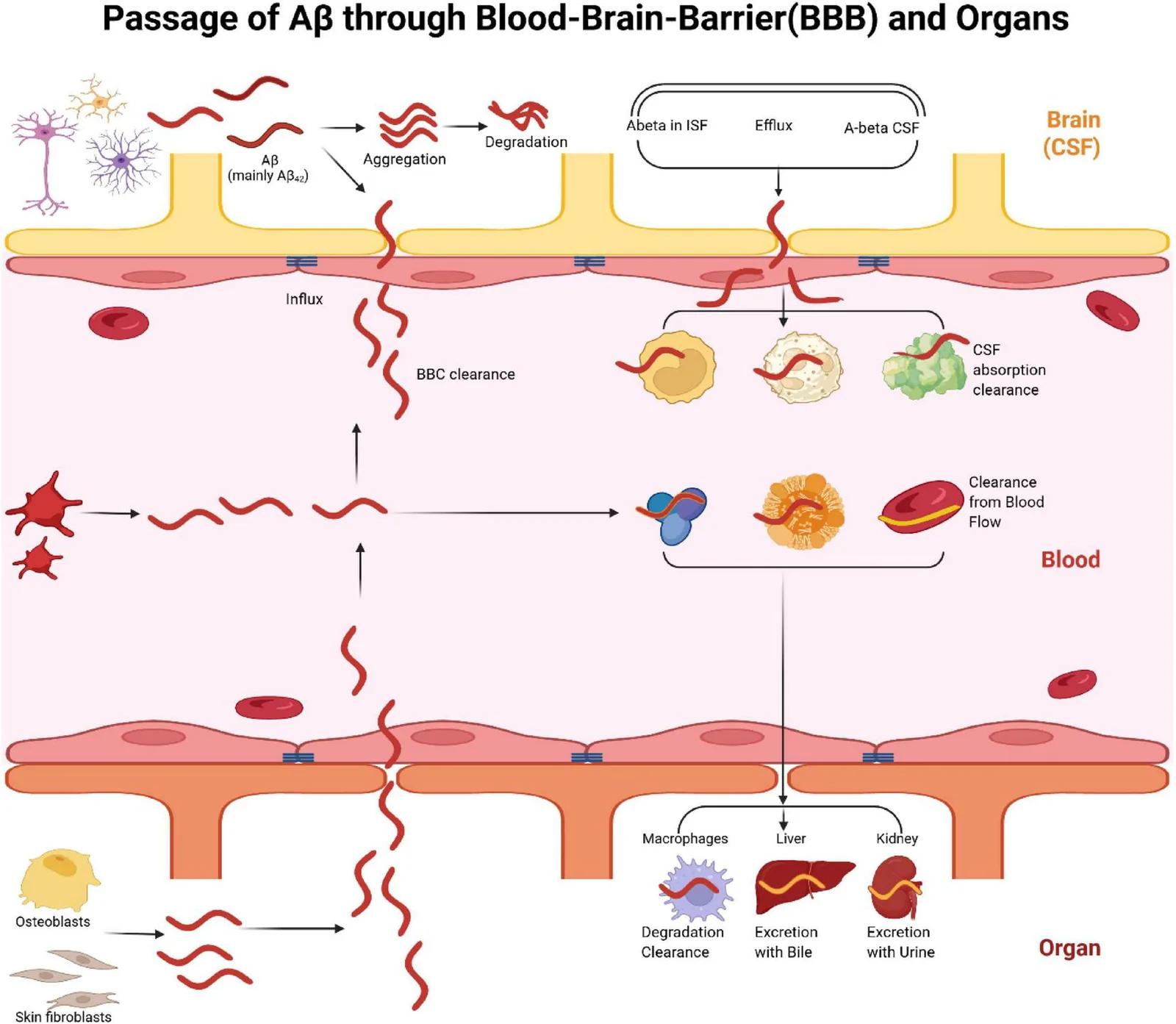
Clearance of Aβ across the blood-brain barrier and peripheral organs. Routes by which Aβ (predominantly Aβ42) is cleared from the brain and processed peripherally. In the brain, neuronally derived Aβ undergoes aggregation and enzymatic degradation; soluble Aβ in the interstitial fluid (ISF) is effluxed into the cerebrospinal fluid (CSF) and removed through cellular uptake and CSF-absorption clearance. Aβ also crosses the BBB bidirectionally (influx and efflux) between brain and blood. Once in the circulation, Aβ is cleared from the bloodstream and transported to peripheral organs, where it is degraded by macrophages or excreted via the liver (biliary excretion) and kidneys (urinary excretion). Peripheral sources such as osteoblasts and skin fibroblasts also contribute to the systemic Aβ pool. Impaired clearance through these central and peripheral routes contributes to Aβ accumulation in AD. Created in https://BioRender.com.

Peripheral myeloid cells, lymphocytes, oligodendrocytes, and astrocytes play a role in neuroinflammation associated with AD, apart from the activation of vascular cells, leading to a leak in the BBB and involvement of the glymphatic system (Figures 3, 4). Clinical studies are currently testing new therapeutic strategies that target the inflammatory aspects of AD. Research is also focused on these pathways for future experiments (Heneka et al., 2025).

### Cerebellar changes in AD

The molecular alterations observed in the cerebellum during AD include Aβ accumulation, neurofibrillary tangle formation, chronic neuroinflammatory activation, heightened OS, and metabolic dysregulation, which together promote Purkinje neuron degeneration and progressive cerebellar atrophy (Hampel et al., 2021).

A distinct subgroup of microglia associated with neurological dysfunction is characterized by activation of the integrated stress response (ISR), a conserved cellular stress signaling pathway (Flury et al., 2025). Independent activation of ISR genes in microglia promotes the early emergence of so-called “dark microglia,” which are linked to synapse loss and disease progression. In AD models, microglial ISR activation exacerbates synaptic loss and neurodegeneration, whereas ISR suppression mitigates these pathological effects. ISR activation further compromises neuronal survival and stability, in part by inducing the release of neurotoxic lipids from microglia. Pharmacological inhibition of either lipid production or ISR signaling reduces synapse loss in AD models, although ISR-driven toxicity may persist partially through sustained lipid-mediated mechanisms.

### RORα

**FIGURE 5:**
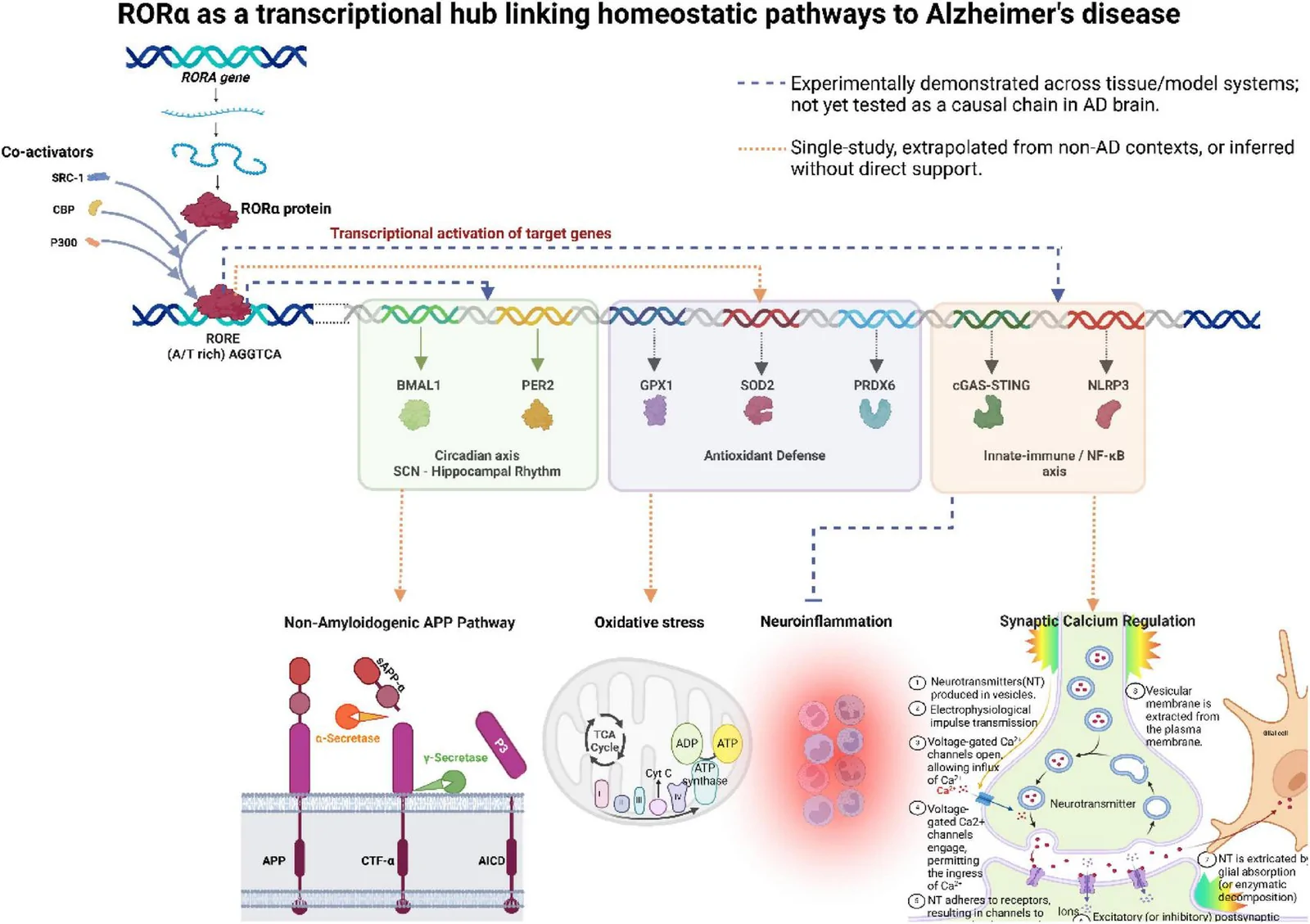
RORα as a transcriptional hub linking homeostatic pathways to AD. This overview summarizes the organizing framework of the review. RORα binds ROR response elements (RORE; an A/T-rich sequence followed by an AGGTCA half-site) as a monomer and recruits the co-activators SRC-1, CBP, and p300 to drive transcription of target genes across three homeostatic axes: a circadian axis (BMAL1, PER2; SCN-hippocampal rhythm), an antioxidant-defense axis (glutathione peroxidase 1 (GPX1), superoxide dismutase 2 (SOD2), peroxiredoxin 6 (PRDX6); mitochondrial redox balance and mitophagy), and an innate-immune/NF-κB axis (cyclic GMP-AMP synthase-stimulator of interferon genes (cGAS-STING), NLR family pyrin domain-containing 3 (NLRP3)). Through these mechanisms, RORα is proposed to influence downstream processes implicated in AD, including non-amyloidogenic APP processing, synaptic calcium regulation, oxidative-stress resistance, and neuroinflammation. Created in https://BioRender.com.

RORα is part of the unique monomeric orphan nuclear receptor NR1 subfamily of nuclear hormone receptors (NHRs). These receptors can function as key transcription regulators. They have several ways to achieve strong and selective DNA binding (Giguère et al., 1995). It regulates transcriptional activation and repression and plays a vital role in the development of the cerebellum (Harding et al., 1997; Figure 5). The focus on the elements of docosahexaenoic acid in ablation of *RORA* takes place during cerebellar development (Chen et al., 2020).

### RORα is a pleiotropic transcription factor

RORα (NR1F1; gene *RORA*) is a member of the nuclear receptor superfamily and shares the canonical six-region (A-F) modular architecture of these receptors (Jetten, 2009). The N-terminal A/B region carries a ligand-independent activation function (AF-1); the C region is a highly conserved DNA-binding domain (DBD) built from two zinc-finger motifs; the “D region” is a flexible hinge; the “E region” is the ligand-binding domain (LBD), which houses the ligand-dependent activation function-2 (AF-2); and the short, variable F region lies at the C-terminus. Unlike receptors that bind DNA as heterodimers with RXR, RORα engages DNA as a monomer: the DBD, aided by a C-terminal extension, recognizes a ROR response element (RORE) comprising a single PuGGTCA [(A/G)GGTCA) core motif preceded by a 5’ A/T-rich extension (Giguère et al., 1994; Jetten, 2009).

Transcriptional output at ROREs is set by the LBD. RORα is constitutively active; structural and biochemical work indicates that cholesterol and cholesterol sulfate act as proposed agonists, whereas 7-oxygenated sterols (e.g., 7α-hydroxycholesterol, 7-ketocholesterol) function as inverse agonists that bind the LBD and dampen activity (Wang et al., 2010b). Agonist occupancy stabilizes helix 12/AF-2 in the active position, forming the hydrophobic surface that recruits LXXLL-motif coactivators: SRC-1 (NCOA1) and related p160 coactivators, the histone acetyltransferases CBP and p300, and PGC-1α (Jetten, 2009). Repositioning of helix 12 instead exposes an overlapping surface for the CoRNR-box corepressors NCoR (NCOR1) and SMRT (NCOR2), which recruit HDAC3 and impose a deacetylase-dependent repressive state (Jetten, 2009). RORα therefore operates as a ligand-tunable switch between activation and repression at a single class of response element and isoform diversity broadens this output. The human *RORA* locus generates four isoforms, RORα1 - RORα4, through alternative promoter usage and 5’ exon splicing; they share an identical DBD and LBD but differ in their N-terminal domains, which fine-tune DNA-binding preference and transactivation (Giguère et al., 1994; Park et al., 2019). RORα1 and RORα4 are conserved in both mouse and human and are broadly distributed RORα1 across many tissues and RORα4 predominating in liver whereas RORα2 and RORα3 are human-specific and restricted to a narrow set of tissues (Park et al., 2019).

RORα is a pleiotropic transcription factor, and its activity spans at least five physiological programs. In circadian regulation, RORα is a constitutive component of the core clock. It binds ROREs in the *Bmal1* (*Arntl*) promoter to drive rhythmic transcription and opposes the repressor REV-ERBα in the stabilizing limb of the transcription–translation feedback loop. Loss of RORα dampens *Bmal1* oscillation and destabilizes daily locomotor rhythms (Akashi and Takumi, 2005; Sato et al., 2004; Jetten, 2009). In metabolism, RORα regulates cholesterol and lipid handling, hepatic glucose and bile-acid pathways, adipose and bone homeostasis, and thermogenic programming, partly through transcriptional crosstalk with PPAR signaling, and its dysregulation is associated with steatohepatitis and dyslipidemia (Hams et al., 2020; Han et al., 2014; Serra et al., 2006). In immunity, RORα directs T-helper-17 differentiation together with RORγt and separately restrains innate inflammatory tone by acting as a negative regulator of NF-κB, tuning macrophage activation, and helping maintain intestinal and tissue immune homeostasis (Delerive et al., 2001; Nejati Moharrami et al., 2018; Oh et al., 2019; Yang et al., 2008). This immunomodulatory role is context-dependent rather than uniformly anti-inflammatory, as discussed below. In endocrine signaling, RORα is itself hormonally regulated: thyroid hormone controls its expression in the developing cerebellum, and its neuroprotective effects depend in part on systemic sex-steroid status (Boukhtouche et al., 2006c; Janmaat et al., 2011; Koibuchi and Chin, 1998). In neuronal development, RORα is required for cerebellar maturation. It directly drives the Purkinje-cell gene program (*Pcp2*, *Pcp4*, *Itpr1*, *Shh*, *Slc1a6*) and dendritic differentiation, and its loss produces the staggerer phenotype of Purkinje and granule-cell degeneration (Chen et al., 2013; Gold et al., 2003; Harding et al., 1997). This range of circadian, metabolic, immune, endocrine, and neurodevelopmental functions is the basis for considering RORα a multi-pathway node in AD, and it is also why augmenting RORα carries a risk of off-target transcriptional effects.

Across these isoforms, RORα is expressed in cerebellar Purkinje cells and other CNS regions, skeletal muscle, skin, adipose tissue, liver, and immune compartments, where it governs Purkinje-cell development and survival, circadian rhythm, lipid and bone metabolism, and the suppression of inflammatory signaling (Jetten et al., 2013; Jetten, 2009). In the cerebellum, RORα directly drives a Purkinje-cell gene program that includes Pcp2, Pcp4, Itpr1, Shh, and Slc1a6 (Jetten, 2009), a circuit of direct relevance to the neurodegenerative context developed below.

### RORα in neurodevelopment

RORα is a ligand-activated member of the nuclear receptor family that serves as a transcription factor indispensable for diverse phases of central nervous system formation (Jetten, 2009). Via the control of programs of gene expression that constitute neuronal lineage specification, synaptic assembling, circadian rhythmicity and cellular protective regulation, RORα supports the appropriate cortex organization and cerebellar development during embryonic and postnatal developmental stages (Gold et al., 2003). Alterations in RORα expression or signaling have been associated with impaired neurodevelopment and an increased susceptibility to developmental neurological disorders, underscoring its important role in the establishment and maintenance of normal brain structure and function (Chen et al., 2013). Within NHR superfamily, transcriptional regulators such as peroxisome proliferator-activated receptors (PPARs) and RORα coordinate metabolic, inflammatory and neuronal survival processes that are increasingly implicated in AD pathophysiology (Boukhtouche et al., 2006a). RORα operates as a convergent regulatory node promoting neuronal robustness, particularly within cerebellar networks where it directs Purkinje neuron maintenance and spatial organization across the lifespan (Boukhtouche et al., 2006a). Loss-of-function mutations in *RORA*, as characterized in staggerer mouse models, induce progressive degeneration of Purkinje and granule cell populations accompanied by pronounced cerebellar shrinkage and disruption of cytoarchitecture, reinforcing its pivotal role in transcriptional process that safeguards neuronal stability (Hamilton et al., 1996).

### RORα in neuronal health and survival

The loss-of-function data below derive from *staggerer* and haplo-insufficient models outside the AD context and establish RORα’s role in maintaining baseline neuronal integrity (Lau et al., 2008). Loss-of-function studies underscore the necessity of *RORA* for neuronal health and survival. The importance of *RORA* to the survival and well-being of neurons is demonstrated by the loss-of-function studies. Animal models of *RORA* haploinsufficiency cause Purkinje cell degeneration and cerebellar atrophy which is progressive showing that loss of *RORA* function partially is lethal to the nervous system (Janmaat et al., 2011). The depletion of *RORA* genetically worsens behavioral deficits and cognitive impairment and reinforcing *RORA* elevation restores these phenotypes and neurological functioning, hence supporting the causal correlation between *RORA* levels and neurological functioning (Warhaftig et al., 2021).

**FIGURE 6:**
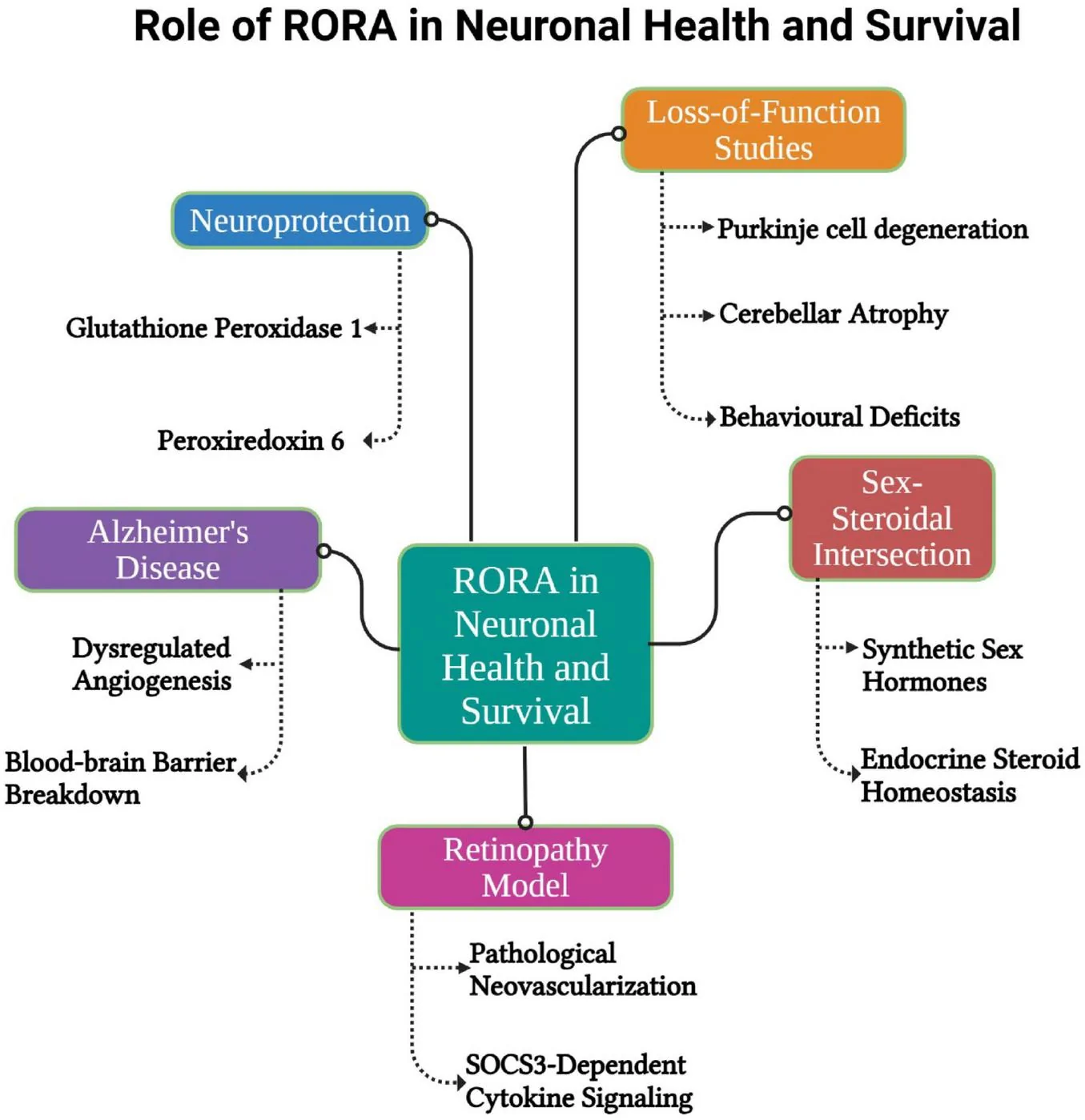
Complex regulatory roles of *RORA* in the neurological and vascular homeostasis. The diagram summarizes the role of *RORA* in a number of cellular mechanisms and disease models: *Neuroprotection (Blue)*: It depicts the role of *RORA* in the resistance of OS by the regulation of Glutathione Peroxidase 1 and Peroxiredoxin 6. *Loss-of-Function Studies (Orange):* This expounds the phenotypic features of *RORA* deficiency that include Purkinje Cell Degeneration, Cerebellar Atrophy, and Behavioral Deficits. *Alzheimer Disease (Purple)*: This is the association of the *RORA* dysfunction with the vascular anomalies, especially with the Dysregulated Angiogenesis and Compromised BBB Integrity. *Sex-Steroidal Interaction (Red)*: This highlights the relationship existing between *RORA* and systemic sex hormones with respect to the maintenance of endocrine steroid homeostasis. *Retinopathy Model (Pink)*: This shows the mechanism by which *RORA* controls pathological neovascularization by Suppressor of Cytokine Signaling 3 (SOCS3)-dependent cytokine activation. Created in https://BioRender.com.

The viability of aging Purkinje neurons depends chiefly on circulating sex steroids rather than locally synthesized neurosteroids, and in *RORA* haplo-insufficient (*RORA*^+^/sg) mice premature depletion of systemic sex hormones markedly accelerates age-related Purkinje cell loss, indicating a functional interaction between RORα signaling and endocrine steroid homeostasis (Boukhtouche et al., 2006a; Figures 5, 6).

AD is marked by dysregulated, excessive but non-productive angiogenic remodeling, evidenced in human post-mortem cortex and *in-vivo* imaging by increased endothelial proliferation and vascular endothelial growth factor (VEGF) signaling concomitant with blood–brain barrier breakdown, pericyte loss, and impaired cerebral perfusion rather than effective vascular regeneration (Iturria-Medina et al., 2016). In this context, *RORA*-driven inflammatory angiogenic programs, as revealed in retinopathy models, may similarly promote aberrant cerebrovascular remodeling in AD via suppressor of cytokine signaling 3 (SOCS3)-dependent cytokine signaling, thereby coupling vascular dysfunction to neuroinflammation and impaired amyloid clearance (Sun et al., 2015).

Neuroprotective ability of *RORA* in neurodegenerative disease models has also been supported by further investigation undertaken using experimental techniques. It has been shown that despite the well-established activity of *RORA* in antioxidant response and cellular resistance mediators, amplified *RORA* expression attenuates amyloid-mediated neuronal toxicity by triggering key redox-defense enzymes, in particular, the GPx1 and PRDX6 (Boukhtouche et al., 2006a). Furthermore, pharmacological activation of *RORA* using the selective agonist SR1078 provides significant neuroprotection against toxin-induced neuronal death, suggesting that *RORA* modulation represents a viable therapeutic strategy (Al-Zaid et al., 2023). Taken together, these findings drawn largely from non-AD neurodegeneration models suggest that increasing *RORA* activity genetically or pharmacologically may be neuroprotective, a possibility that remains to be tested directly in AD (Figure 6).

**FIGURE 7:**
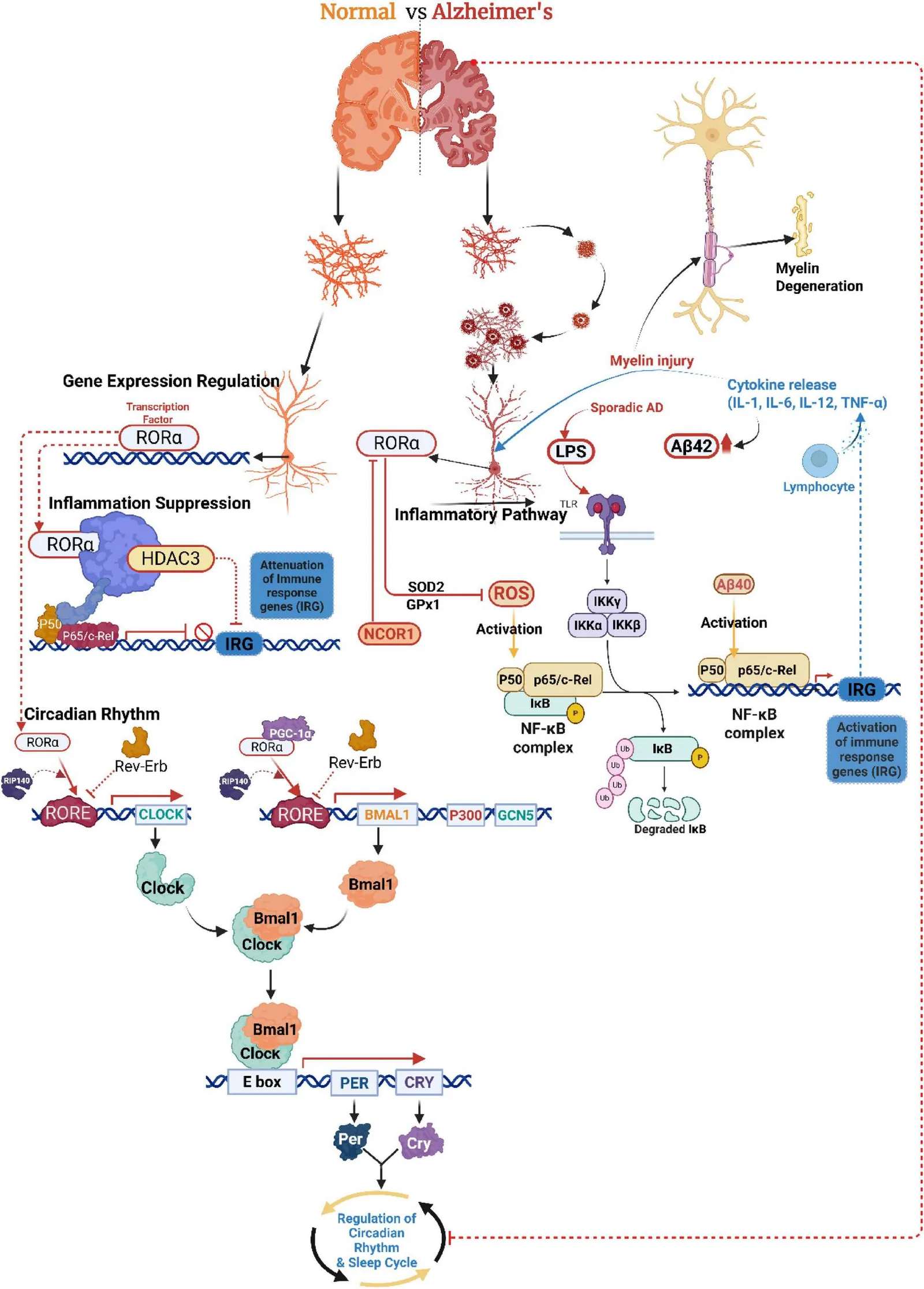
Expression and functional analysis of *RORA* in Normal vs Alzheimer’s brain. Under normal conditions, in a healthy neuronal cell, RORα acts as a transcription factor that positively regulates genes related to the circadian rhythm and inflammation suppression. Role of comparative molecular signaling of *RORA* in neuroprotection and AD-related degeneration. The comparison of homeostatic and pathological signaling is detailed in a schematic: *Normal State (A)*: *RORA* is also a transcriptional regulator which regulates inflammation inhibition by the recruitment of histone deacetylase 3 (HDAC3) and maintenance of the circadian rhythm *in-vivo* by interacting with CLOCK/BMAL1 complexes. *AD State (B)*: When subjected to pathological stimulus (lipopolysaccharide (LPS), aggregated Aβ42) the NF- kB complex becomes activated that results in release of proinflammatory cytokines (IL-1, IL-6, TNF) that leads to episodic myelin degeneration. *Mitigation*: How the down regulation of immune-response genes (IRGs)/up-regulation of antioxidant enzymes (SOD2, GPx1) mediated through RORα. Created in https://BioRender.com.

In AD, dysregulated activation of NF-κB signaling occurs through signal transduction via a sequence of biochemical reactions and inflammatory responses that ultimately culminate in the activation of immune-related genes, collectively intensifying neuroinflammatory processes. The cytokines (IL-1, IL-6, IL-12, and TNF-α) are released by immune cells, which bring about the myelin injury, leading to myelin degeneration. LPS triggers NF-κB mediated increases in cytokines, which raise Aβ levels. This process induces oligodendrocyte injury and leads to myelin destruction, which is a characteristic of AD. It works by acting on the leukocyte and microglial Toll-like receptor 4 (TLR4)-CD14/TLR2 receptors (Zhan et al., 2018). AD’s asymptomatic stage disrupts the circadian clock, which may elevate neurodegeneration. Within the suprachiasmatic nucleus (SCN), RORα acts to activate BMAL1 transcription. The core clock in the SCN controls RORα, which is needed for proper BMAL1 expression and the consolidation of daily locomotor activity (Sato et al., 2004). RORα reduced the amount of reactive oxygen species (ROS) by increasing the activity of enzymes that remove ROS, specifically GPx1 and SOD2 (Han et al., 2014; Figure 7).

Beyond this antioxidant role, RORα also acts upstream of the NF-κB-driven inflammatory pathway described above. RORα binds directly to ROR response elements and, together with the corepressor NCoR1 and histone deacetylase 3 (HDAC3), controls transcription of NF-κB-dependent immune response genes (Oh et al., 2019). Because this control occurs at the promoter level, RORα sits upstream of the inflammatory gene output, not downstream of it. In astrocytes, this upstream control is state-dependent: RORα represses IL-6 transcription in the resting state but permits or increases IL-6 transcription once astrocytes become reactive (Journiac et al., 2009). This shows that RORα’s upstream regulatory function changes with the inflammatory state of the cell, rather than acting as a fixed switch. RORα also acts upstream of the core circadian clock. It binds ROR response elements in the Bmal1 promoter and directly activates Bmal1 transcription, in competition with Rev-Erb repression at the same sites; loss of RORα destabilizes this rhythm (Akashi and Takumi, 2005). This positions RORα as an upstream driver of circadian gene expression, independent of its role in inflammation. *RORA* expression is altered in AD tissue: hippocampal transcriptomic data show *RORA* upregulated in AD and placed within gene networks that also contain APP (Acquaah-Mensah et al., 2015). This association places *RORA* within the same regulatory network as amyloid-related genes, but the data do not establish that RORα drives APP expression or Aβ production; the direction of that relationship is not resolved by current evidence. On this basis, RORα is best described as an upstream regulator of inflammatory and circadian gene transcription, acting in parallel to Aβ and tau rather than upstream of their generation.

### RORα in neuroinflammation

*RORA* plays a broader role in immune regulation by reducing inflammatory responses and controlling the inflammatory state of human macrophages (Hams et al., 2020). In the brain, microRNA-7 cooperates with RORα to negatively regulate neuroinflammatory processes, further underscoring RORα’s role as a key modulator of inflammation in neural tissues (Yue et al., 2020). Through transcriptional regulation of genes involved in energy metabolism and redox balance, *RORA* activation counteracts convergent mechanisms that drive neuronal vulnerability in neurodegenerative conditions, including AD (Jetten, 2009). Pharmacological accessibility of RORα further supports its modulation as a disease-modifying therapeutic approach capable of stabilizing neuronal function rather than merely alleviating symptoms (Solt et al., 2012). Obese mice’s fat tissue macrophages show higher RORα expression. Myeloid cells without RORα had fewer inflammatory macrophages. This suggests that in metabolic diseases, RORα controls inflammation and macrophage activation (Hams et al., 2020). Recent single-cell transcriptomic analyses demonstrate marked regional and cell type-specific molecular heterogeneity in AD, with the entorhinal cortex exhibiting substantially greater transcriptional dysregulation than the prefrontal cortex. These observations suggest that transcriptional regulators, including RORα, are unlikely to exert uniform effects throughout the diseased brain. Supporting the importance of cellular context, Journiac et al. (2009) demonstrated that in primary astrocytes, pro-inflammatory cytokines induce RORα expression, which directly transactivates the IL-6 promoter. This finding contrasts with the predominantly anti-inflammatory functions attributed to RORα in other experimental systems and indicates that its effects on neuroinflammatory signaling are highly dependent on cell type, regional microenvironment, and disease stage (Lee et al., 2021; Li et al., 2022; Nejati Moharrami et al., 2018; Wang et al., 2021).

### RORα-mediated neuroprotection and the mitophagy-immune checkpoint

**FIGURE 8:**
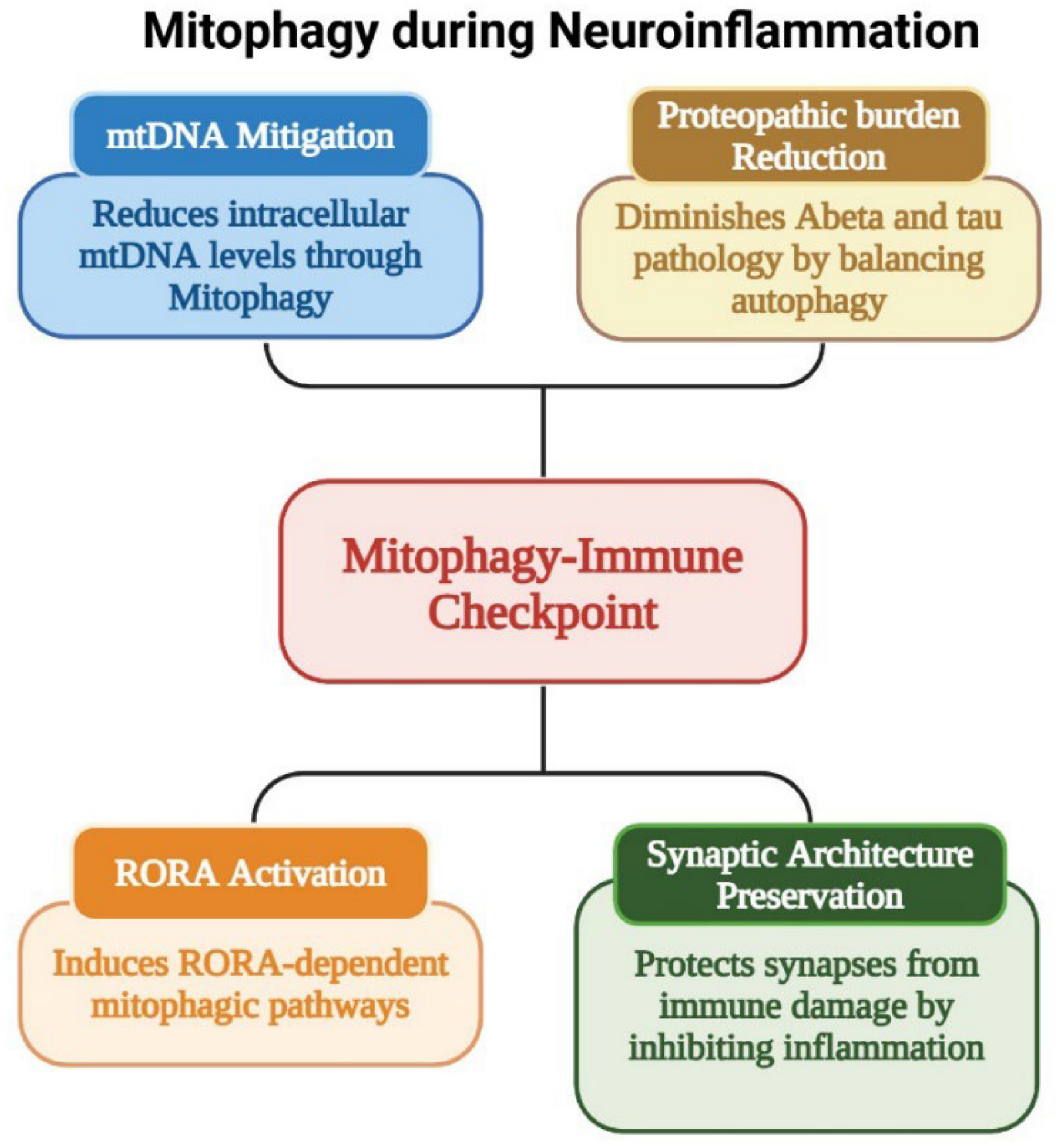
Mitophagy during neuroinflammation: the mitochondrion-immune checkpoint (MIC). Mitochondrion-Immune Checkpoint (MIC) in neuroinflammatory control. The processes by which neuronal integrity is maintained through the mitophagic pathways in the case of inflammatory stress: *Pathology Mitigation:* Regains the balance of autophagy, followed by the decrease of the levels of mitochondrial DNA (mtDNA) and proteopathic burden (including Aβ and tau). *RORA Activation*: MIC activates *RORA*-sensitive processes of mitophagy required for strengthening cell defenses. *Neuroprotection*: By inhibiting inflammation, immune destruction of synaptic architecture is prevented. Created in https://BioRender.com.

The mitophagy-immune checkpoint is characterized chiefly in cardiomyocyte and hypoxic-ischemic systems; its relevance to AD is inferential and bears on the amplification of neuroinflammation rather than its origin (Wang et al., 2020; Xu et al., 2024). RORα has recently been characterized as a central upstream regulator of mitophagy, the targeted autophagic removal of impaired mitochondria. RORα exerts this function through transcriptional control of effectors including caveolin-3, thereby promoting the elimination of dysfunctional mitochondrial populations that would otherwise intensify oxidative burden (Beak et al., 2021; Figure 8).

*The Mitophagy-Immune Checkpoint*: A pivotal advance in understanding RORα function has been the delineation of a mitophagy immune regulatory checkpoint. Through mitochondrial quality control systems involving mitophagic mechanisms, RORα reduces intracellular accumulation of mtDNA. Under neurodegenerative conditions, including AD, escape of mtDNA into the cytoplasm triggers activation of the cyclic GMP-AMP synthase-stimulator of interferon genes (cGAS-STING) innate immune signaling cascade (Song et al., 2025) alongside NOD-like receptor family pyrin domain containing 3 (NLRP3) inflammasome pathways in microglial cells, thereby driving sustained neuroinflammatory escalation. This occurs through processes such as: (a) inflammatory modulation (b) reduction of proteopathic burden (c) through preservation of synaptic architecture through inhibition of NF-κB mediated proinflammatory cytokine production and protection against immune-driven synaptic damage, *RORA* supports the maintenance of precisely organized synaptic connectivity (Delerive et al., 2001; Figure 8).

### RORα’s role in AD

As outlined previously, RORα functions as a central transcriptional coordinator of cerebellar maturation by harmonizing signaling networks among Purkinje neurons, granule cells, and inter-neuronal populations. Cell viability, anti-inflammatory responses regulation and neuronal differentiation are modulated through activation of antioxidant gene expression by RORα through an integrative regulation (Serra et al., 2006). Initial molecular profiling established RORα as the dominant receptor isoform expressed in cerebellar tissue, where it governs Purkinje neuron-specific transcriptional circuits critical for structural development and sustained cellular homeostasis (Koibuchi and Chin, 1998). A single-nucleus multiome analysis of the human AD/ADRD cerebellum placed *RORA* within disease-associated gene-regulatory networks, most prominently in Purkinje cells, with trajectory analysis of granule-cell populations also identifying *RORA* as a disease-relevant transcription factor (Cheng et al., 2024) (Preprint). Experimental loss of *RORA* activity, as observed in staggerer mouse models, produces profound cerebellar underdevelopment characterized by extensive degeneration of Purkinje and granule neurons, culminating in motor impairment, cognitive dysfunction, and stereotyped behaviors that parallel cerebellar-associated deficits documented in AD pathology (Chen et al., 2020).

*RORA* exhibits significantly elevated expression within the hippocampus of individuals in AD (Acquaah-Mensah et al., 2015). Network analyses revealed that a substantial subset of genes showing differential expression in the AD hippocampus were connected to *RORA* (Acquaah-Mensah et al., 2015). Within the AD hippocampus, network analysis identified multiple genes co-associated with *RORA*, including APP, DNM1L, and TIA1, all of which show altered expression in AD (Acquaah-Mensah et al., 2015). These findings represent network-level co-associations rather than experimentally validated transcriptional targets of RORα (Acquaah-Mensah et al., 2015). APP through proteolytic processing produces peptides of Aβ, which accumulate to form extracellular amyloid plaques in AD (Chen et al., 2017). DNM1L (DRP1) has been implicated in AD pathogenesis through dysregulation of mitochondrial dynamics. Aβ and phosphorylated tau physically interact with DRP1, promoting excessive mitochondrial fission, mitochondrial dysfunction, synaptic impairment, and neuronal degeneration (Manczak and Reddy, 2012). TIA1, an RNA-binding protein, regulates tau pathophysiology through interactions that coordinate stress granule formation and RNA metabolism. TIA1 knockdown or knockout inhibits tau misfolding and associated toxicity in cultured hippocampal neurons, while overexpressing TIA1 induces tau misfolding and stimulates neurodegeneration (Vanderweyde et al., 2016). This bidirectional relationship suggests that elevated TIA1 in AD hippocampus (Acquaah-Mensah et al., 2015) may promote rather than suppress tau pathology (Apicco et al., 2018; Vanderweyde et al., 2016). Transcriptomic analyses of genetic variants affecting transcription factor binding distinct from previously reported AD-associated gene expression profiles identified RORα as a differentially expressed TF with approximately threefold increased expression (Dharshini et al., 2019). Although *RORA* is elevated in the AD hippocampus (Acquaah-Mensah et al., 2015; Dharshini et al., 2019) and RORα confers resistance to oxidative damage in dopaminergic systems (Al-Zaid et al., 2023), direct quantitative measurement of *RORA* in the hippocampus and cortex of AD model systems such as APP/PS1 transgenic mice is still lacking.

As discussed above, a key factor in AD is ongoing neuroinflammation (Thakur et al., 2023). By acting as a transcription factor for genes related to inflammation resolution, RORα manages immune responses (Delerive et al., 2001). In the CNS, RORα exhibits cell type-specific inflammatory functions. In primary astrocytes, pro-inflammatory cytokines induce RORα expression, and RORα directly transactivates the IL-6 promoter, increasing IL-6 expression. These findings indicate that the effects of RORα on neuroinflammatory signaling are context dependent rather than uniformly anti-inflammatory (Journiac et al., 2009). While no AD model has directly tested whether RORα deficiency leads to increased microglial activation, elevated Aβ burden, and neurodegeneration, existing non-AD data suggest this hypothesis is experimentally plausible. *RORA* deletion in human THP-1 macrophages triggers constitutive increases in NF-κB-regulated genes TNF, IL-1β, and IL-6 at both mRNA and protein levels, persisting even without LPS stimulation (Nejati Moharrami et al., 2018). Similarly, staggerer mice, which carry a loss-of-function mutation in the RORα gene, exhibit macrophage hyperproduction of IL-1β following LPS stimulation (Kopmels et al., 1992). Critically, this evidence derives from systemic macrophages rather than brain-resident microglia, and from non-neuroinflammatory disease contexts. Whether RORα-dependent regulatory mechanisms in peripheral innate immunity translate to microglial behavior during amyloid and tau accumulation remains unestablished. Direct experimental evaluation requiring *RORA* deletion in microglial-selective AD models such as APP/PS1 transgenic mice, coupled with quantitative assessment of both inflammatory cytokine signatures and pathological Aβ/tau burden is needed to test this mechanism in relevant brain tissue (Nejati Moharrami et al., 2018). In innate myeloid cells, rather than neurodegeneration models, support this inflammatory axis, as genetic loss of *RORA* in human THP-1 derived macrophages and primary murine bone-marrow derived macrophages enhances NF-κB pathway activation and markedly increases pro-inflammatory cytokine production, including IL-1β and TNF-α, following lipopolysaccharide stimulation (Nejati Moharrami et al., 2018). *In-vivo*, myeloid-specific *RORA* deletion in mice alters cytokine responses during endotoxemia, demonstrating that *RORA* directly tunes inflammatory amplitude in macrophages through NF-κB-linked transcriptional programs (Nejati Moharrami et al., 2018). RORα modulates innate immune activation by regulating NF-κB dependent inflammatory programs in macrophages, where loss of *RORA* enhances chromatin accessibility at cytokine loci and increases expression of pro-inflammatory mediators including interleukin-1β and tumor necrosis factor-α following immune stimulation (Hams et al., 2021; Nejati Moharrami et al., 2018). Given the conserved transcriptional architecture of macrophages and microglia, and the central role of NF-κB signaling in driving microglial cytokine release and neurotoxic inflammation in neurodegenerative disease, RORα-dependent control of inflammatory amplitude likely extends to microglial reactivity in neuroinflammatory contexts (Heneka et al., 2015; Heppner et al., 2015). Although the *RORA* levels were significantly increased in the cerebral tissue of people diagnosed with AD, the effect of this upregulation is most probably the efforts of a neuroprotective mechanism in support of compensation on chronic NF-κB-mediated neuroinflammation, and not the driving force of the pathological process. RORα may also act in a context-dependent manner, much as some PPARs are anti-inflammatory in one tissue while regulating other pathways elsewhere; its net effect likely depends on the local microenvironment and available co-factors. On this view, the elevated hippocampal *RORA* in AD reflects a compensatory response to sustained inflammation rather than a driver of pathology.

The activation of the microglia of AD also leads to an increase in expression levels of proinflammatory cytokines (Huang and Mahley, 2014). The release of pro-inflammatory mediators by these toxified microglia intensifies the Aβ aggregation and neuron death (Doens and Fernández, 2014). It has therefore been found that persistent microglial stimulation is one of key pathogenesis of AD (Noh et al., 2025).

### RORα and OS

**FIGURE 9:**
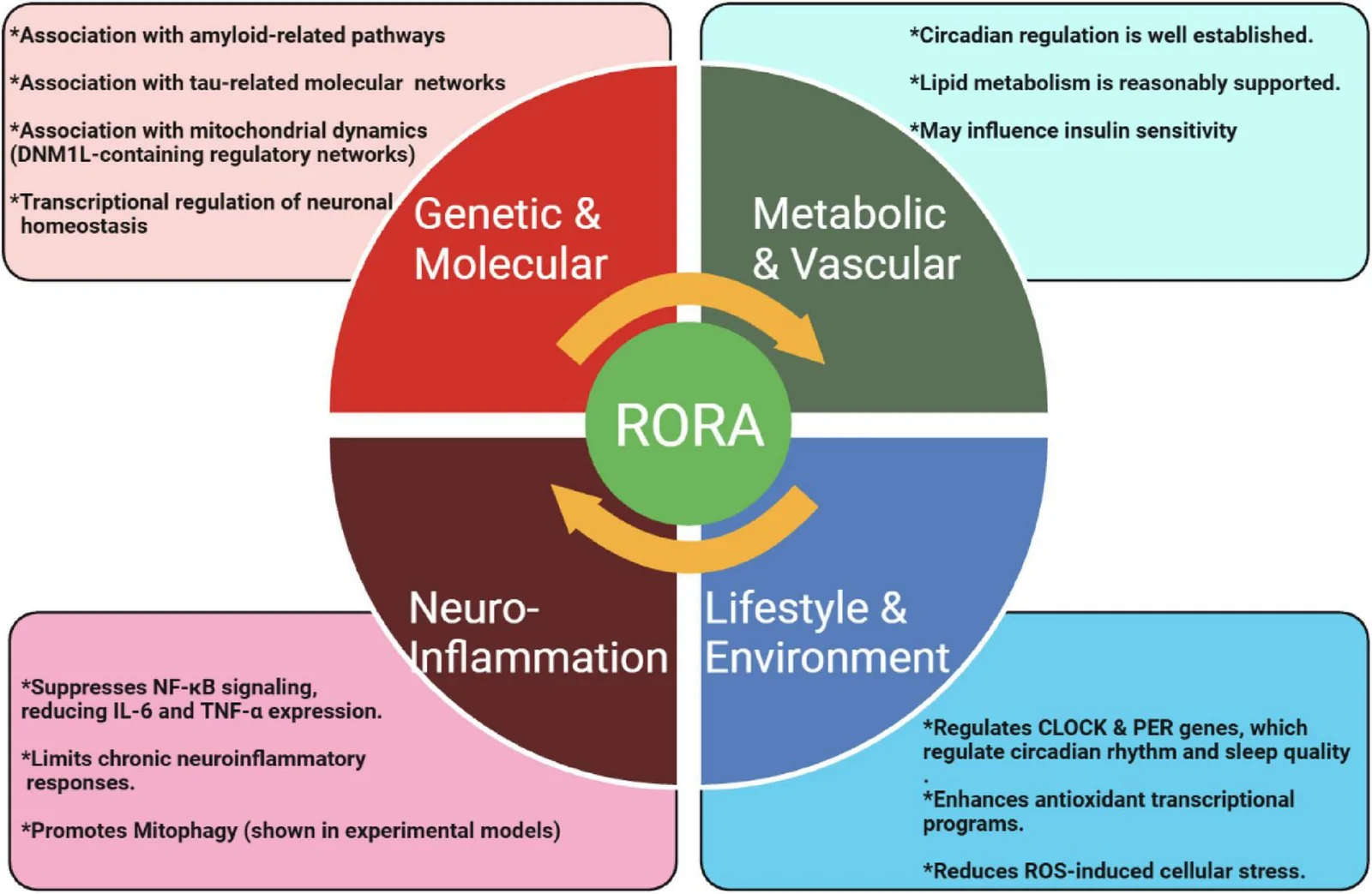
Risk Factors associated with AD and the possible role of *RORA*. Key modulatory action of *RORA* in attenuating multifactorial risk factors of AD. Here, the multiple protective responses of *RORA*: Genetic and Molecular: Modulation of APP transcription and inhibition of Aβ and Tau aggregation. Metabolic and Vascular: Enhancement of Insulin Receptor sensitivity and Lipid Metabolism (e.g., APOE). Neuroinflammation: Suppression of NF-kappaB to inhibit chronic inflammation and promote Mitophagy. Lifestyle and Environmental: Regulation of Circadian Rhythm (CLOCK/PER) genes and reduction of ROS Accumulation. Created in https://BioRender.com.

The antioxidant functions described here are drawn from dopaminergic, hepatic (NASH) and related non-AD systems and concern baseline redox homeostasis running alongside, rather than through, the amyloid-tau cascade. An imbalance in the production of reactive oxygen species (ROS) or the loss of antioxidant defenses disturbs cell redox homeostasis triggering OS which only increases with the age of the brain (Andreyev et al., 2005). Analysis of post-mortem human substantia nigra tissue and dopaminergic cell culture studies have identified RORα as a key regulator of neuroprotective responses against OS (Al-Zaid et al., 2023; Figure 9).

This pathway overlaps with mechanisms implicated in the early synaptic dysfunction and heightened neuronal susceptibility characteristic of AD. Besides, experimental evidence shows that *RORA* and its cognate ligands inhibit hepatic inflammation and OS to alleviate the development of nonalcoholic steatohepatitis (NASH). Collectively, these observations highlight the therapeutic potential of *RORA* based interventions for neurodegenerative conditions driven by oxidative injury, including AD (Al-Zaid et al., 2023). OS in conjunction with mitochondrial impairment constitutes an early, self-amplifying cascade that promotes synaptic degeneration, Aβ cytotoxicity, and neuronal loss in AD. Within this disease framework, the capacity of *RORA* signaling to restrain inflammatory pathways and oxidative damage in NASH models supports the concept that evolutionarily conserved, *RORA*-dependent cytoprotective mechanisms regulating redox balance may be exploited to counteract OS-mediated neurodegeneration (Han et al., 2014).

### RORα in circadian rhythm regulation (CRR) and hippocampal function

The involvement of RORα in AD pathology extends to CRR and hippocampal function, both of which are severely disrupted in AD patients. The 3 × Tg-AD mouse carries the human APP^Swe, PS1^M146V and tau^P301L mutations and develops progressive amyloid and tau pathology alongside early circadian and sleep-wake disturbances resembling those in AD. In this model, hippocampal *RORA* mRNA levels correlate positively with Aβ and tau burden, demonstrating that hippocampal RORα expression increases concomitantly with pathological burden rather than showing compensatory downregulation (Bellanti et al., 2017). In the suprachiasmatic nucleus, by contrast, circadian clock-gene expression, including the RORα-BMAL1 limb, is disrupted in AD models (Yang et al., 2025). Given that circadian disruption exacerbates Aβ accumulation and cognitive decline, restoring RORα-mediated CRR may provide indirect neuroprotective benefits. Additionally, RORα’s role in hippocampal neurogenesis and synaptic plasticity positions it as a key regulator of memory formation and cognitive function which are compromised early in AD (Acquaah-Mensah et al., 2015). Therefore, in preclinical AD, improving sleep and circadian rhythms may provide an opportunity for early intervention to stop the progression of the disease. RORα is a key regulator of circadian rhythm by directly activating BMAL1 transcription through ROR response elements (ROREs) in the BMAL1 promoter. Perturbation of RORα disrupts circadian gene expression and downstream metabolic homeostasis (Akashi and Takumi, 2005; Maglione et al., 2015). Altered RORα activity perturbs PPAR signaling through transcriptional crosstalk among nuclear receptor pathways which changes the lipid metabolic processes governing lipid storage, mobilization, and utilization (Jetten, 2009; Figure 9).

### Pharmacological modulation of RORα

RORα is constitutively active, and much of its basal transactivation appears to depend on a lipid occupant of the ligand-binding pocket rather than on an inducible hormone (Kallen et al., 2002). Crystallographic and biochemical work points to cholesterol and cholesterol sulfate as the endogenous occupants that support this activity, though their status as bona fide physiological ligands has never been fully settled (Jetten, 2009; Wang et al., 2010b). Oxysterols bind the same pocket with defined, isoform-specific consequences: 25-hydroxycholesterol serves as the standard radioligand in RORα binding assays and functions as an inverse agonist for RORα (suppressing activity), while functioning as an agonist for RORγ (promoting activity). The 7-oxygenated derivatives 7α-hydroxycholesterol and 7-ketocholesterol function as inverse agonists for both RORα and RORγ (Wang et al., 2010b). The receptor is therefore best understood as tonically active and tunable downward by its own sterol environment.

SR1078, an early synthetic agonist built on the T0901317 scaffold, activates both RORα and RORγ and raises expression of ROR target genes including glucose-6-phosphatase and FGF21 (Wang et al., 2010a). Nobiletin, a citrus polymethoxyflavone, binds the RORα and RORγ LBDs directly and acts as an agonist, amplifying circadian oscillation through RORE-dependent induction of Bmal1; its affinity is higher for RORγ than for RORα, and its clock and metabolic effects are abolished when RORα/γ are knocked down (He et al., 2016).

T0901317 was among the first small molecules shown to repress RORα and RORγ; however, it is a liver X receptor agonist first and foremost, and its mixed pharmacology precludes isolation of ROR-specific effects. SR1001 and SR3335 represent more selective RORα-targeted inverse agonists developed to overcome this limitation (Kumar et al., 2010). SR3335 (ML-176) was developed from that scaffold as the first RORα-selective inverse agonist, binding RORα with a Ki near 220 nM, displacing 25-hydroxycholesterol from RORα but not RORγ, and suppressing hepatic gluconeogenic target genes (Kumar et al., 2011). SR1001, a dual RORα/RORγt inverse agonist (Ki = 172 and 111 nM, respectively), overcomes the LXR polypharmacology of T0901317. It shifts the LBD toward corepressor recruitment, suppresses IL-17 transcription, and blocks Th17-driven autoimmunity in mice (Solt et al., 2011).

Potent, selective, brain-penetrant agonists capable of raising RORα function in neurons do not exist in a form suitable for chronic CNS use. That asymmetry is a large part of why restoring or augmenting RORα in the nervous system is more realistically achieved by gene delivery than by pharmacology, the argument developed in the section that follows.

## Gene therapies

Gene therapy can make up for lower levels of bioactive chemicals by directly boosting enzyme activity which happens by delivering the transgene using a vector and is usually a virus that infects the host cells. The gene is then expressed inside these cells. Gene therapy has been successfully used to treat several neurological and neurodegenerative conditions (Daci and Flotte, 2024).

## *RORA* as a potential gene therapy candidate

The feasibility of *RORA* based gene therapy has recently been demonstrated through proof-of-concept studies using adeno-associated virus vectors. Akula et al. (2024) have shown how a murine model of Stargardt disease has been successfully treated using the *RORA* gene with an adeno-associated viral (AAV) vector with considerable down-regulation of the APP expression accompanied by RORα expression into retinal cells (Akula et al., 2024). Though the main aspect of the study was on the retinal degeneration, the attenuation of APP as one of the elements in the pathology of AD suggests a future potential in the translation of such evidence to a clinical AD setting; however, further empirical validation is required to generalize the results to a medical AD scenario (Akula et al., 2024). The AAV-*RORA* approach demonstrated good tolerability and efficient transduction of retinal tissue (Akula et al., 2024), providing translational evidence that *RORA* gene augmentation is technically achievable and biologically effective in modulating AD-relevant pathways. These insights support AAV-*RORA* genetic intervention as an appealing candidate for additional investigation of potential clinical improvement in Alzheimer management.

**FIGURE 10:**
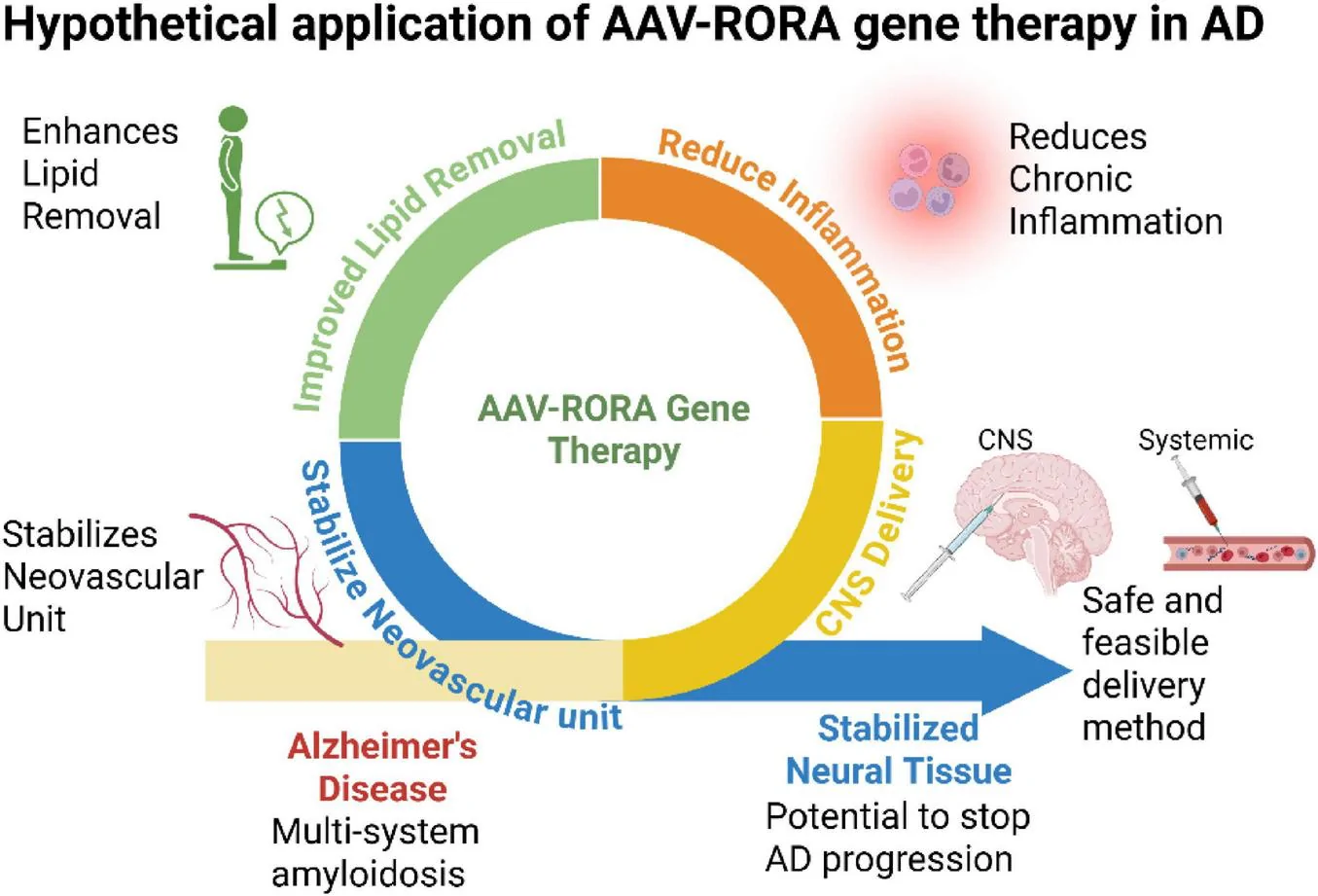
Proposed Mechanistic Framework for *RORA*-Based Therapeutic Modulation in AD. This theoretical framework illustrates the potential of *RORA* augmentation as a disease-modifying strategy to slow AD progression by targeting multiple pathogenic pathways. Implementation would require targeted CNS delivery (e.g., intrathecal or brain-region-specific AAV injection) to achieve the desired cell-type specificity. Created in https://BioRender.com.

Given the absence of direct evidence for RORα deficiency in AD models and the context-dependent bidirectionality observed in non-neuroinflammatory systems, we propose that RORα should not be framed as a global therapeutic target. Instead, any consideration of RORα augmentation would require region- and cell-type-restricted approaches, confined to regions and cell populations in which a functional RORα deficit has been experimentally demonstrated. As discussed earlier, *RORA* is reported to be upregulated in the AD hippocampus; whether this pattern generalizes to other AD-vulnerable brain regions remains unknown (Acquaah-Mensah et al., 2015). A cell-type-specific assessment of *RORA* expression in the AD hippocampus using single-nucleus RNA sequencing (snRNA-seq) would be critical for defining the cellular localization and directionality of *RORA* dysregulation. Such analyses are necessary because bulk tissue measurements can obscure divergent changes across distinct cell populations, potentially masking biologically relevant effects. Moreover, the documented bidirectional functions of RORα, including pro-inflammatory activity in microglia and neuroprotective effects in neurons, indicate that the consequences of dysregulation are likely highly context dependent. Finally, the rationale for any therapeutic strategy targeting *RORA* would require evidence of a cell-type-specific and potentially reversible deficit, thereby identifying the appropriate cellular target and direction of intervention. Should future studies identify a cell-type-specific *RORA* deficit, therapeutic approaches would prioritize selective neuronal or glial targeting (e.g., synapsin-1, GFAP promoters) to isolate beneficial effects from potential off-target effects. The fact that the local microenvironment influences RORα, and RORα itself acts as a negative regulator of the immune system, possibly through various downstream pathways (Haim-Vilmovsky et al., 2021; Singh et al., 2023), makes it a potential candidate for gene therapy. *RORA* has been tested as a gene therapy candidate in mice (Singh et al., 2023). Recent research indicates that genetic intervention using viral vectors can improve RORα activity, lessen the OS as well as improve neuronal survival. AAV-based delivery of the *RORA* gene has emerged as a therapeutic strategy under investigation due to its demonstrated capacity to confer neuroprotection and sustain cognitive function (Singh et al., 2023). In Abca4^–/–^ murine models of Stargardt disease and dry age-related macular degeneration (AMD), administration of AAV5-hRORA produced several beneficial effects, including a reduction in subretinal deposit accumulation (Akula et al., 2024), normalization of CD59 expression involved in complement regulation, suppression of APP levels (Akula et al., 2024) and enhancement of photoreceptor performance (Akula et al., 2024; Figure 10).

All these findings highlight RORα’s role in regulating the inflammation processes, the activity of the complement system, and neurodegenerative mechanisms, and define its application as a regulator of gene-therapy strategies (Singh et al., 2023). This entails the use of AAV-mediated human *RORA* delivery to treat dry age-related degeneration of the macula and Stargardt disease. The molecular overlap with neurodegenerative pathways, particularly complement activation and inflammation, identifies RORα as a potential target of central nervous system applications (Singh et al., 2023). Processes that are commonly dysregulated in AD include inflammation (Oh et al., 2019), circadian rhythm regulation (Guillaumond et al., 2005), and neuroprotection (Al-Zaid et al., 2023), which are often controlled by RORα. Being a transcription factor, *RORA* controls anti-inflammatory functional genes and synaptic wellness, such as those of microglial action and lipid metabolism (Hams et al., 2021). Even though the exact role of *RORA* in the microglial activity in the AD models is yet to be completely clarified, its proven anti-inflammatory properties and mitochondrial integrity regulation indicate that a depletion of *RORA* could exaggerate the effect of the microglial response (Song et al., 2025), which would inhibit A-beta clearance and promote neurodegeneration. Since RORα is a regulator of numerous pathways, which are involved in AD, it is emerging as a useful target of gene-therapy interventions. Normal transcriptional activity, decreased inflammation, and the prevention of additional neuronal injury may be achieved through AAV-based delivery which restores the expression of *RORA* (Daci and Flotte, 2024). Modifier gene therapy based on *RORA* holds promise to be gene-agnostic, which may be important given the complex etiology of diseases involving inflammation, activation of complement, and OS where activation of many pathogenic mechanisms at once can be seen. There is potential for diseases involving the CNS, especially in Alzheimer because of its role in regulating APP, CD59 (Akula et al., 2024), and inflammatory cytokines (Journiac et al., 2009) as observed from studies in other neurodegenerative disorders. This strategy offers a potential new way to change the course of the disease rather than just addressing visible symptoms, opening doors for more tailored treatments in AD.

## Investigational *RORA* therapy for degenerative disorders

OCU410 is an investigational gene therapy that utilizes an AAV5 vector encoding the human *RORA* gene (AAV5-hRORA) (Ocugen, 2025). It is designed in a way that modulates a repertoire of biological pathways involved in the retinal degeneration process including inflammatory response pathways, complement system detection, lipid metabolism, OS, and photoreceptor homeostasis. Preclinical studies in *Abca4^–/–^* murine models of Stargardt disease and dry age-related macular degeneration demonstrate that OCU410 reduces subretinal deposit accumulation, normalizes complement regulator CD59, suppresses APP expression, and protects photoreceptor function (Akula et al., 2024).

OCU410 is currently under clinical investigation for retinal degenerative diseases, where its single-administration AAV platform is designed to deliver sustained *RORA* expression to retinal tissue (Shah et al., 2025). These ongoing clinical trials are trying to provide evidence of the intervention in relation to safety, tolerability and bio-efficacy in human retinal pathologies, thus creating hope that someday there will be therapeutic modalities.

Given that the *RORA* gene and RORα protein regulate inflammation, OS, mitochondrial integrity, complement pathways, and neuroprotection mechanisms also implicated in AD the applicability of OCU410 in an AD context is scientifically plausible. The molecular overlap between retinal degeneration and AD pathology, including shared inflammatory and complement-driven processes, suggests that OCU410 may have potential relevance beyond ocular disease.

Future work should test these effects in AD-relevant neurons, examining circuit dysfunction, neuroinflammation, and amyloid and tau pathology in both *in-vitro* and *in-vivo* models. Consequently, while the biological rationale is compelling, OCU410 remains investigational, and its extension into AD therapeutics requires rigorous future study.

Nonetheless, supplementary studies are warranted to investigate whether AAV5-mediated *RORA* delivery could present therapeutic advantages in AD. Significant subsequent actions comprise investigating its ramifications on AD-specific neuronal populations, circuit-level dysfunction, neuroinflammation, and amyloid/tau-associated pathology in pertinent *in-vitro* and *in-vivo* research designs.

## Future directions

### *RORA*: translational potential for CNS disorders

*RORA* regulates (i) APP (Akula et al., 2024; Yang et al., 2025) CD59 (complement inhibitor) (Akula et al., 2024) (iii) functional differentiation of T cells (T helper 17) and IL-17 expression (Yang et al., 2008), IL-6, TNF-α, and phosphorylation of STAT3, and (iv) BDNF (brain-derived neurotrophic factor) (Acquaah-Mensah et al., 2015). Such parallels in metabolic perturbation of AD make it a compelling target central nervous system intervention including its use in Parkinson and amyotrophic lateral sclerosis, and other neuroinflammatory diseases. *RORA*’s function supports the wider idea of the modifier gene therapy paradigm, which is: 1. Mutation-independent; 2. Pathway-focused; 3. Scalable across diseases.

Considerable evidence exists to justify the hypothesis that inflammation is a targetable therapeutic intervention in AD (Wang et al., 2015). Long-term use of non-steroidal anti-inflammatory drugs (NSAIDs) has been proposed to lower AD risk, but controlled trials of anti-inflammatory agents including NSAIDs, minocycline, PPAR-γ agonists, and TNF-α–targeting drugs have largely failed to show benefit (Calsolaro and Edison, 2016).

Although prior studies have identified increased *RORA* expression in the hippocampal compartments of AD models and implicated it in OS protection, direct quantitative characterization of *RORA* within cerebellar AD model systems, including APP/PS1 transgenic mice, has not been performed (Al-Zaid et al., 2023; Hampel et al., 2021).

The reported changes in *RORA* expression across AD models, and the gaps that remain, are summarized in Table 1.

**TABLE 1:** Reported *RORA*/RORα expression changes across brain regions in human Alzheimer’s disease tissue and transgenic mouse models.

System	Model type	Region	Direction of *RORA*/RORα change	References
Human AD, post-mortem transcriptomic network analysis	Human AD tissue	Hippocampus	Increased; *RORA* a network hub (insulin/BDNF)	(Acquaah-Mensah et al., 2015)
Human AD brain, transcriptome study	Human AD tissue	Hippocampus	Increased ≈3-fold (differentially expressed TF)	(Dharshini et al., 2019)
Human AD/ADRD, snRNA-seq + snATAC multiome	Human AD tissue	Cerebellum (Purkinje cells; granule-cell trajectory), Frontal cortex	Regulatory network/cCRE association; direction not quantified. Preprint	(Cheng et al., 2024)
3 × Tg-AD (APP^Swe/PS1^M146V/tau^P301L)	Transgenic mouse	Hippocampus	RORα mRNA positively correlates with Aβ/tau (not a WT-vs-AD fold change)	(Bellanti et al., 2017)
APP^Swe/PS1dE9	Transgenic mouse	SCN, hippocampus, cortex	*Rora* rhythm/expression altered across regions	(Yang et al., 2025)
APP/PS1	Transgenic mouse	Cerebellum	Not determined, no direct *RORA* measurement	Absence noted (Al-Zaid et al., 2023; (Hampel et al., 2021)

Direction of change is reported as measured in each cited study; human post-mortem findings reflect end-stage disease, whereas transgenic-model findings capture defined pathological stages. Hippocampal *Rora* in 3 × Tg-AD is reported as a correlation with Aβ/tau burden rather than a genotype-based fold change. Cheng et al. is a preprint at the time of writing, and its *RORA* association is network- and chromatin-based rather than quantified expression change. Abbreviations: ADRD (AD-related dementias); cCRE (candidate cis-regulatory element); SCN,(suprachiasmatic nucleus); snATAC-seq (single-nucleus assay for transposase-accessible chromatin sequencing); snRNA-seq (single-nucleus RNA sequencing).

Such investigations are required to determine whether cerebellar *RORA* downregulation occurs in association with Aβ and tau pathology and whether this dysregulation contributes causally to cerebellar neurodegeneration in AD (Yang et al., 2020).

The fact that anti-inflammatory medications target general rather than specific neuroinflammatory components in AD may contribute to these unclear results. Therefore, in order to clarify the possibility of curbing neuroinflammation at the early phases of the disease, there is a dire need to establish the definite modulators of inflammatory processes that work at the initial stages.

## Discussion

Here we discuss RORα as a transcriptional regulator integrating inflammatory, oxidative, synaptic and metabolic homeostasis across AD-vulnerable regions, including the hippocampus, suprachiasmatic nucleus and cerebellum. Although the cerebellar Purkinje-cell evidence is among the most direct, we note that cerebellar pathology is a comparatively late and minor feature of AD; the cerebellar findings are therefore presented as one supporting strand of a broader regional hypothesis rather than the principal therapeutic rationale (Gold et al., 2003). This convergence suggests RORα as a candidate target for cerebellar dysfunction associated with AD that warrants direct testing (Gold et al., 2003). In lieu of concentrating on specific pathological features, RORα augmentation provides a systems-level approach designed to restore endogenous neuron stability and circuitry (Jetten, 2009). Systems of transcriptional reprogramming are also beginning to be viewed as effective systems to manipulate sophisticated neurodegenerative phenotypes (Deverman et al., 2018). In retinal models and early clinical studies, *RORA* delivery reduces toxic lipid accumulation, dampens complement-mediated inflammation and attenuates OS (Khanani et al., 2026; Trakkides et al., 2019).

These pathological processes parallel those observed in the AD cerebellum, where lipid debris, chronic neuroinflammation and redox imbalance contribute to Purkinje cell dysfunction and degeneration (Mavroudis et al., 2019; Trakkides et al., 2019). Shared molecular programs between retinal neurons and Purkinje cells, including expression of PCP2 and PCP4, further support cross-CNS translational relevance (Gold et al., 2003).

In retinal models, *RORA* is positioned as an upstream regulator of protective pathways: *RORA* loss permits downstream accumulation of toxic lipids, complement activation, and inflammatory cascades (Akula et al., 2024). Whether *RORA* occupies the same upstream position in AD acting as a preventive transcriptional brake rather than solely as a resilience factor remains to be established through direct AD-model studies. Resolving this distinction is therefore a critical precursor to a rational therapeutic strategy.

Augmentation of *RORA* represents a network-level intervention capable of restoring intrinsic neuronal stability and circuit function rather than targeting isolated downstream pathologies (Acquaah-Mensah et al., 2015; Boukhtouche et al., 2006b; Chen et al., 2013; Delzor et al., 2012; Mathiesen et al., 2020). Mechanistically, *RORA* has been implicated, largely in non-AD systems, in several homeostatic programs spanning inflammatory repression, synaptic maintenance and OS resistance (Gold et al., 2003; Gold et al., 2007; Han et al., 2014). RORα antagonizes NF-κB signaling through induction of IκBα and direct interference with inflammatory transcriptional complexes, thereby suppressing IL-1β and IL-6 expression (Nejati Moharrami et al., 2018). RORα concurrently regulates calcium signaling and synaptic stability genes including ITPR1 and PCP4 that are essential for Purkinje dendritic architecture and parallel fiber integration (Gold et al., 2003; Gold et al., 2007). Restoration of these pathways supports the potential for structural and functional cerebellar circuit recovery rather than solely arresting neuronal loss. RORα further enhances intrinsic antioxidant defenses through transcriptional induction of enzymes such as GPX1 and SOD2 (Han et al., 2014; Figure 5). These effects are potentially relevant to AD, where Aβ oligomers are thought to contribute to synaptic dysfunction in part through ROS generation and calcium dysregulation (De Felice et al., 2007). Purkinje cells in AD cerebella exhibit dendritic degeneration and synaptic abnormalities consistent with these stress mechanisms (Mavroudis et al., 2019). Collectively, these data support a model in which Purkinje-specific *RORA* augmentation can restore homeostatic transcriptional networks, stabilize cerebellar circuitry and enhance neuronal resilience within a persistently pathological environment (Boukhtouche et al., 2006b; Gold et al., 2003; Mattson, 2004; Figure 5). This resilience-oriented framework is conceptually complementary to amyloid-targeting strategies rather than an alternative to them. Anti-amyloid immunotherapies have produced the first disease-modifying signals in AD, whereas RORα augmentation has no clinical data in AD; if validated, a transcriptional-resilience approach might address pathways those therapies do not, potentially in combination, rather than supplanting them (De Felice et al., 2007; Mattson, 2004). Key translational considerations include maintenance of physiological RORα expression levels, optimization of cerebellar vector distribution and validation of functional rescue in disease-relevant models (Fischell and Fishman, 2021; Nitta et al., 2017). Single-cell transcriptomic approaches combined with electrophysiological and behavioral readouts provide powerful platforms to confirm network reprogramming and circuit restoration following gene therapy. Together, existing mechanistic data and emerging clinical precedent position *RORA* gene augmentation as a biologically plausible candidate strategy whose translatability remains to be established for cerebellar involvement in AD (Gold et al., 2003). Transcriptional hub modulation has emerged as a promising strategy for complex neurodegenerative disorders characterized by multi-pathway collapse (García-González et al., 2024).

## Limitations, risks, and safety considerations of *RORA* augmentation

A central limitation of any RORα augmentation proposal is the incomplete regional mapping of *RORA* expression in AD. Bulk transcriptomic data place *RORA* above control levels in the hippocampus, yet whether this pattern extends to or diverges in other AD-vulnerable regions (entorhinal cortex, prefrontal cortex, cerebellum) remains unknown. Additionally, bulk measurements cannot assign these changes to specific cell types, obscuring potentially opposing effects across neurons, glia, and immune cells (Acquaah-Mensah et al., 2015). A prerequisite for any *RORA*-targeted therapeutic strategy whether global or restricted is demonstration of a functional *RORA* deficit in AD brain. Current evidence does not establish *RORA* deficiency in any AD-vulnerable region or cell type. As discussed earlier, the hippocampal *RORA* is upregulated; whether this reflects pathogenic activation, compensatory response, or bystander dysregulation remains unresolved. Without evidence of a correctable deficit, *RORA* augmentation cannot be justified. Resolving this requires cell-type-resolved snRNA-seq of AD hippocampus, validated in situ by RNAscope, to determine the direction of the *RORA* change in each population; that experiment, rather than further modeling, is the gating step for a defensible therapeutic design.

Off-target effects and supraphysiological expression are important risks for any RORα augmentation strategy. A rational therapeutic design should therefore ensure both regional targeting and cell-type restriction. In principle, cell-type selectivity could be achieved using cell-type-specific promoters (e.g., synapsin-1 for neurons, GFAP for astrocytes) combined with region-restricted delivery; however, no published studies have demonstrated this approach for *RORA* in cerebellar populations. Such promoter control would limit augmentation to the cellular contexts in which benefit is intended. This restriction would also reduce ectopic upregulation in neurons already expressing elevated *RORA*. While overexpression of regulatory proteins can disrupt transcriptional networks, the specific effects of *RORA* overexpression have not been characterized (Yang et al., 2020). Should systemic delivery be considered, CNS-tropic AAV serotypes such as AAV9/PHP.eB would preferentially target brain tissue while reducing peripheral transduction. However, this approach would require cell-type-specific promoters to maintain cellular restriction (Deverman et al., 2018). A therapeutic strategy would require demonstrating a correctable *RORA* deficiency in specific cerebellar populations through cell-type-resolved snRNA-seq. If such a deficit were identified and confirmed distinct from hippocampal *RORA* elevation, selective cerebellar targeting with cell-type-restricted promoters would minimize off-target effects in *RORA*-elevated populations. *RORA*’s pleiotropic effects regulating inflammation, circadian rhythms, lipid metabolism, and neuronal survival create both therapeutic opportunity and safety risk. Benefit in one cell type could be offset by toxicity in another if off-target expression occurs. The potential limitations and the risk mitigation strategies are outlined below.

*1. Astrocyte inflammation:* In astrocytes, pro-inflammatory cytokines induce RORα, and RORα in turn directly transactivates the IL-6 promoter and raises IL-6; in already-activated astrocytes the same receptor instead restrains IL-6 through NF-κB, so the direction of effect is state-dependent rather than uniformly anti-inflammatory (Journiac et al., 2009). That unresolved bidirectionality is the concern: augmenting RORα in a brain undergoing astrogliosis alters neuroinflammatory tone in a direction current data cannot predict, and because IL-6 is neurotrophic or neurotoxic depending on context. This risk can be mitigated by first establishing the cell state–specific effects of RORα in reactive astrocytes using human single-cell and disease-relevant preclinical models, and by employing regulated, cell-selective expression systems that enable precise control of RORα activity while monitoring downstream IL-6 and neuroinflammatory signaling.

*2. Immune gain-of-function*: RORα is required, alongside RORγt, for full Th17 differentiation, and enforced expression promotes rather than merely permits the Th17 program (Yang et al., 2008); it is also essential for ILC2 (nuocyte) development and type 2 immunity (Wong et al., 2012). Vector biodistribution to lymphoid tissue, or leak from a CNS route into the periphery, could therefore drive Th17 and ILC2-mediated inflammation, converting a neuroprotective intent into an autoimmune or allergic liability. Restricting RORα expression to the intended CNS cell populations through targeted vector design, immune-cell detargeting strategies, and localized delivery approaches that minimize exposure of lymphoid tissues and reduce the potential for Th17- or ILC2-mediated peripheral immune activation.

*3. Systemic and metabolic effects*: RORα regulates hepatic gluconeogenic genes, cholesterol and bile-acid handling, and lipid storage, and its loss produces dyslipidemia and altered fat metabolism (Jetten, 2009; Lau et al., 2008); the selective inverse agonist SR3335 lowers hepatic gluconeogenic targets, confirming these outputs are RORα-responsive (Kumar et al., 2011). AAV delivered systemically, or redistributing from the CNS, transduces liver efficiently, so metabolic perturbation is a foreseeable off-target consequence of anything short of tightly contained delivery. This limitation could be mitigated by restricting transgene expression to the target CNS cell population through localized delivery, CNS-tropic vectors with minimal peripheral biodistribution could reduce the risk of off-target metabolic effects.

*4. Region specificity and delivery:* RORα’s functions are cell-type- and region-specific, from Purkinje-cell survival in the cerebellum to the distinct programs it runs elsewhere (Jetten, 2009), so uniform overexpression risks disrupting local balance in regions where its level is already appropriate. This risk can be overcome by using regionally targeted delivery approaches, cell-type-specific and regulatable expression systems, and AAV capsids with restricted tropism to achieve therapeutic *RORA* modulation only in the affected neuronal populations while preserving physiological expression in unaffected brain regions.

Taken together, these points reframe *RORA* augmentation as a matter of controlled, restricted expression rather than simple restoration. The multi-pathway reach that motivates the approach is inseparable from its capacity for *off-target* and *gain-of-function* harm, and the preclinical program should treat isoform selection, spatial containment, dose, and the inflammatory and circadian consequences of constitutive activity as primary design questions rather than downstream refinements.

## Conclusion

In summary, the evidence presented positions RORα as a biologically plausible, network-level regulator of inflammatory, redox, synaptic, and circadian programs relevant to AD across vulnerable regions including the hippocampus, suprachiasmatic nucleus and cerebellum. However, the case rests largely on indirect and non-AD data, and the directional inconsistency of RORα expression across brain regions remains unresolved.

The decisive next step is direct functional testing in region-specific RORα manipulation in established AD models with amyloid, tau, and cognitive read-outs before any therapeutic claim can be made. Investigational retinal AAV-RORα programs make such CNS testing technically conceivable, but translation to the brain remains unproven. RORα is therefore best regarded at present as a promising hypothesis for AD rather than a validated therapeutic target.

Taken together, these points reframe RORα augmentation as a matter of controlled, restricted expression rather than simple restoration. *RORA’s* pleiotropic effects on inflammation, circadian rhythms, and metabolism create risks of off-target toxicity and unintended pathway activation. Preclinical development must address isoform selection, spatial containment, dose titration, and quantification of inflammatory and circadian effects before construct design.
